# 

**Published:** 1889-01-19

**Authors:** 


**Notice to Advertisers and Subscribers.**
All Advertisements, Orders for Copies of THE HOSPITAL, and Business Communications should be addressed to The Publisher, *not to the Editor*, THE HOSPITAL, 38, Old Jewry, E.C.

**To Residents, Secretaries, and Matrons.**
The Editor will be glad to have the Regulations and each Report as issued by Asylums, Hospitals, Convalescent and Nursing Institutions, as well as those of other Charities, and an early copy in duplicate of all Appeals and Festival Notices, etc., addressed to *The Editor, The Lodge, Porchester Square, London, W.* Any superintendent, secretary, matron, or other chief official who regularly sends such information may be registered, on application to the Publisher, to receive free of cost a cover for binding THE HOSPITAL in half-yearly volumes.
A Scale of Charges will be found on the first page of the NURSING SUPPLEMENT.

### Scraps and Gleanings.

Hospital Sunday at Aberdeen resulted in a collection of over £700.

Dr. Currie has been appointed medical officer of Ballymena Dispensary.

German measles have again broken out in Staffordshire, and the schools are closed.

The foundation-stone of the new Eye and Ear Hospital at Hereford has just been laid.

The workmen's contribution to Leeds Hospital, from J. S. Stock's works, reached £40.

There were forty-five applications for the post of house surgeon to Stockport Infirmary.

The annual ball in aid of the Nottingham Children's Hospital was held on January 8th.

St. Michael's Hospital ball took place at Kingstown on January 8th, and was well attended.

At Whipsnade, near Dunstable, no death has occurred for two years amongst the inhabitants.

The Belfast Dispensary Committee are considering a proposal to establish a district midwife.

The workmen of Mander Brothers, Wolverhampton, have subscribed £19 to the General Hospital.

A ball in aid of Bootle Borough Hospital was held in Bootle Town Hall on Thursday, the 27th ult.

The annual ball in aid of the Italian Hospital took place on January 8th. It was hoped to clear £100.

Manchester Royal Eye Hospital has received a cheque for £1,000 from Mr. Lees, of Werneth Park.

Dr. Irving has been appointed medical officer of No. 10 district, Manchester, in place of Dr. Rains.

The amount of funded capital of the East Suffolk and Ipswich Hospital last year was £26,307 10s. 9d.

The Working Men's Committee of Hull Royal Infirmary lately held a concert for the benefit of that institution.

Two concerts are to be given in aid of Sidcup Cottage Hospital, the first of them to take place on February 7th.

Dr. O'Connor, of Blackrock, is being made the subject of lively discussion by the Cork Dispensary Committee.

Dr. Heslop has been holding ambulance classes for the police of Manchester, and has passed 92 out of 94 men.

The Robertson Stewart Hospital is not overworked. The patients during last year numbered seven, of whom three died.

The cost for in-patients at the Gorleston Cottage Hospital last year was 10s. 2d. a-head, and for out-patients 2s. 4d. a-head.

A new hospital for infectious diseases has been erected at Faversham, in which special attention has been paid to ventilation.

Mrs. Robert Smith, the founder of the Hertford and Benges Nurses' Institution, appeals for subscriptions to clear off a debt of £32.

The annual fête at the Dublin Orthopædic Hospital was doubly successful this year owing to an amusing magic lantern exhibition.

It is proposed to close Winter Street Hospital, Sheffield, and the move would be a wise one. It is not a popular hospital by any means.

A special meeting of the Leicester Provident Dispensary members has been convened to investigate the charges against the management.

The friendly societies of the North are warmly taking up the convalescent home scheme. One Durham district has voted it £15.

A very large and successful ball in aid of Cumberland Infirmary was held on January 3rd. It is hoped the receipts will amount to £200.

An amateur dramatic performance in aid of the City of Dublin Hospital was recently given in the Gaiety Theatre to a large audience.

The annual entertainment of the nurses, servants, and children of Mrs. Hilton's Crèche, an orphanage in Stepney Causeway, took place on the 29th ult.

The Secretary of Bristol Hospital for Sick Children and Women thanks those who have subscribed the necessary funds for the new Nurses' Home.

The Queen has sent a donation of £50 to the Children's Aid and Refuge Fund, 32, Charing Cross, for the rescue of destitute and neglected children.

The workpeople of Messsr. J. S. Fry and Sons have, during the last year, contributed £313 12s. 7d. to the Bristol, Bath, and Weston-super-Mare medical charities.

Dr. Riva reports the case of a woman, aged thirty, who was wholly blind in one eye. She suffered from a carious tooth on this side, and after its extraction the vision was restored.

The Victoria Home, which has recently been opened in Paris, is meant for the reception of indigent Englishwomen over 68 years of age, who have spent at least thirty years in France.

### Conditions.

I. Every paper sent in must be clearly written, and one side only must be used. All competitors must mark at the head of their papers the number of their competition.

II. Each paper must be signed by the author with his or her real name and address. A *nom de plume* may be added if the writer does not desire to be referred to by us by his real name. In the case of all prizewinners, however, the real name and address will be published.

III. All communications relating to prize competitions should be addressed to "The Prize Editor, The Hospital, 38, Old Jewry, London, E.C." Competitors are requested not to include in letters, etc., to the Prize Editor, communications on matters of other business. All letters must be fully prepaid.

IV. The Prize Editor of The Hospital is the sole judge of the competitions, and his decision is in all cases final and without appeal.

V. The Prize Editor reserves the right to publish any paper sent in, whether it receives a prize or not. He does not hold himself responsible for any MSS. sent, nor can he undertake to return rejected competitions.

### Amusements and Relaxation.

### NEW PUZZLE PRIZE SCHEME.

SECOND QUARTERLY COMPETITION COMMENCED JANUARY 5th, ENDS MARCH 30th, 1889.

1. Prizes will be awarded for the best Original Puzzles in Prose or Verse relating to any of the Social, Political, or Philanthropical topics of the day. Competitors are requested to *strictly* observe this rule, or their contributions will be discredited.
2. Puzzles may take any form which the ingenuity of the competitors suggest. Answers must accompany contributions, and the source of obscure or ambiguous terms be given.
3. No competitor will be allowed to take more than one prize during the Quarter.
4. Eight Prizes of Standard Books will be given, the choice of the book to be left to the winner. First, Second, Third, Fourth, and Fifth Prizes for best Original Puzzles, £1 1s., 15s., 10s. 6d., 7s. 6d., and 5s. each.
5. First, Second, and Third Prize for Solutions of 15s., 10s. 6d., and 5s. each.

N.B.—All contributions must reach the office, 38, Old Jewry, E.C., not later than the *First Post* on *Thursday* in each week.

### PUZZLES FOR SOLUTION.

**Third Week.**

No. 6. Double Acrostic.

1. Warrior and conqueror, still thy fame
Lives in an eastern city's name.
2. Mother, whose many daughters fair;
Men oft invoke for genius rare.
3. Two vast countries I divide,
Many the nations on either side.
4. Columns and caverns in me are found.
The sea washes my shores all round.
5. A favourite he! as histories tell,
Yet like many, through treason fell.
6. A Queen, both wise and good and great,
Who loved her country, improved her state.
7. A writer's name who through her pen,
Won admiration from all men.
8. In me you'll find a singer's name,
Who wedded another of greater fame.
9. Italia! Land of art and song,
This thy dead son, will charm us long.
10. This you surely will obtain,
When this puzzle is made plain.

My primals to the weary one
My finals often give;
And ere these pages you have done,
Then both you will receive. Cotonopolis.

No. 7. Wheel Puzzle.

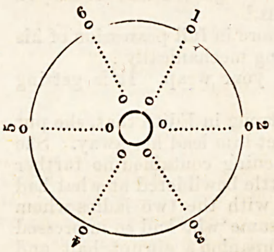

Ever the wheel of life goes round,
And all the treasures it has found
It gives, then takes away,
Two topics it doth bring to-day.
The one is where a siege was laid,
The other is a thing half paid.

1. A decoction or country gives this little word,
A hospital also! whichever's preferred.
2. Of three classes of men, each with different name,
This union, in Turkey, enjoys great fame.
3. A lord of the forest, most gracefully high,
To the pigmies below me, touching the skies.
4. A thousand miles long—of no importance. though—
Ever to the Volga my sluggish waters flow.
5. 'Tis in different languages that you'll find me:
I'm funny, I know, and most peculiar to see.
6. She is not forgotten, though of a time so old,
For her actions were daring, enchanting and bold! Morico.

**Marks for Original Puzzles.**

| Names. | No. of Puzzles sent in Jan. 10. | No. of Marks Jan.10. | Totals. | Names. | No. of Puzzles sent in Jan. 10. | No. of Marks Jan.10. | Totals. |
|---|---|---|---|---|---|---|---|
| Cotonopolis... | 3 | 7 | 7 | Patience ...... | 3 | 6 | 6 |
| Tinie............ | 3 | 6 | 6 | Padre ......... | 1 | 3 | 3 |
| Jenny Wren . | 3 | 4 | 4 | Prior............ | 3 | 2 | 2 |

**Answers.**

No. 1. Double Acrostic.

C uri O

H el P

I mag O

L ai D

B ours E

L ega L

A steroi D

I ag O

N umismati C

No. 2. Conundrum. Because they sent instructions to Su(e) a kin.

No. 3. Diamond Puzzle.

H
E M S
R O S E S
S K A T I N G
H M S T A N L E Y
C O R N E U S
E L L E N
P E N
Y

**Marks for Solution of Puzzles.**

Total Marks.—Flora (1), 1; Mater, 0; Scrooge (1), 1; Patience (0); Lady Clara (2), 2; Ruffles (1), 1. [For Conditions, see preceding column.]

*The Figures in parentheses are Marks for the week ending Jan. 12th.*

